# Community-based interventions for hepatitis C virus elimination among people who use drugs in Shanghai, China

**DOI:** 10.1186/s12954-026-01498-8

**Published:** 2026-07-08

**Authors:** Xiaoxiao Yu, Yingqiu Shen, Jiayi Wang, Guichun Ji, Qiuli Xu, Yumeng Gao, Zhenglan Zhang, Xingyi Chen, Xin Xin, Lihong Qu

**Affiliations:** 1 Department of Infectious Diseases, Shanghai East Hospital, School of Medicine, Tongji University, Shanghai, China; 2 Department of Public Health, Shanghai East Hospital, School of Medicine, Tongji University, Shanghai, China; 3 Beicai Community Health Service Center, Pudong New Area, Shanghai, China; 4 Shanghai Pudong New Area Centre for Disease Control and Prevention, Shanghai, China; 5 Department of Infectious Diseases, Shanghai Pudong Hospital, Fudan University, Shanghai, China; 6 Department of Medical Administration, Shanghai East Hospital, School of Medicine, Tongji University, Shanghai, China; 7 Pudong Institute of Preventive Medicine, Fudan University, Shanghai, China

**Keywords:** Hepatitis C virus, People who use drugs, Direct-acting antivirals, Community

## Abstract

**Background:**

The prevalence of hepatitis C virus (HCV) infection among people who use drugs (PWUD) in China remains high, yet diagnosis and treatment rates are low. Strategies are urgently needed to achieve the World Health Organization’s goal of eliminating HCV as a public health threat by 2030.

**Aim:**

To improve HCV diagnosis and treatment rates among PWUD in Pudong New Area, Shanghai, China.

**Methods:**

A non-randomized controlled intervention study was implemented among individuals with a substance addiction history in Pudong New Area, Shanghai, China. Three models were explored in our study. In the Self-Referral (SR) model, HCV antibody-positive individuals relied on their own initiative to seek medical services. In the Standard Intensive Referral (SIR) model, managerial supervision was strengthened. The Active Intensive Referral (AIR) model placed greater emphasis on managerial proactivity, enhancing screening and treatment for high-risk populations.

**Results:**

A total of 3926 PWUD from 36 communities in Pudong New Area underwent rapid HCV antibody testing in the SR model. These numbers were 1973 and 191, respectively, in the SIR and AIR models. Only 18.9% population completed HCV RNA testing in the SR model compared with 100.0% in both the SIR and AIR models (*P* < 0.001). Overall, 70/146(47.9%) PWUD in the SR model were identified with HCV RNA, consistent with the SIR and AIR models [339/916(37.0%) and 74/191(38.7%), *P* > 0.05]. In terms of treatment, 47/74(63.5%) of patients in the AIR model initiated treatment, significantly higher than the 15/70 (21.4%) in the SR model and 73/339 (21.5%) in the SIR model (*P* < 0.001). Genotype 3, particularly subtype 3b, was the most prevalent genotype among PWUD in our study, respectively accounting for 23/38 [60.5%] in the AIR model and 112/166 [67.5%] in the SIR model. In the AIR model, all participants received a liver stiffness test. Fifty-two (27.2%) patients had a liver stiffness of more than 12.5 kPa. One hundred and eighty-one persons completed the questionnaire in the AIR model. The results revealed that few people had high educational attainment. More than half (52.5%) of the population had no stable marital relationship.

**Conclusions:**

A multidisciplinary community-based approach significantly improved HCV diagnosis and treatment among PWUD. Despite limitations, the AIR model may represent a promising approach to improving the HCV care cascade among PWUD and could potentially contribute to China’s efforts to optimize cascade management for hepatitis C elimination among specific high-risk populations.

## Introduction

Hepatitis C virus (HCV) infection continues to pose a major global public health challenge [1, 2], particularly among people who use drugs (PWUD) [3], a population disproportionately affected by the disease. Despite the advent of direct-acting antivirals (DAAs), which have transformed HCV treatment with cure rates exceeding 95% [4], significant barriers to diagnosis, treatment initiation, and retention in care persist in this vulnerable group [5, 6]. The World Health Organization (WHO) has set an ambitious goal of eliminating HCV as a public health threat by 2030, defined as an 80% reduction in incidence and a 65% reduction in mortality compared to the 2015 baseline [7–9]. Achieving this goal requires innovative and context-specific strategies tailored to key populations.

Globally, PWUD often experience limited access to testing and treatment services due to social marginalization, stigma, and structural barriers such as criminalization and lack of integration between addiction treatment and infectious disease care [10, 11]. Studies from Europe, North America, and Asia have demonstrated that community-based and integrated care models, particularly those incorporating peer support, point-of-care testing, and decentralized treatment, can substantially increase diagnosis and treatment uptake [12, 13]. For instance, interventions in supervised injecting facilities and opioid agonist therapy clinics have proven successful in engaging PWUD in the HCV care cascade [14, 15].

In China, the burden of HCV among PWUD remains substantial, with prevalence rates significantly exceeding those of the general population. Despite the inclusion of highly effective direct-acting antivirals (DAAs) in the National Reimbursement Drug List, treatment uptake in this group remains critically low [16, 17]. In this traditional system, individuals identified in community screenings are expected to navigate the transition to specialized hospital care independently—a pathway frequently obstructed by social stigma, logistical hurdles, and a lack of integrated support between addiction services and clinical hepatology [18, 19]. Currently, there is a lack of evidence-based, multidisciplinary models tailored to the unique social determinants of health found in Chinese PWUD populations, such as low educational attainment and the absence of stable social support systems. Our study addresses this research gap by evaluating three distinct referral intensities in the Pudong New Area of Shanghai. By comparing a Self-Referral (SR) model against a Standard Intensive Referral (SIR) and a newly developed Active Intensive Referral (AIR) model, we aim to demonstrate how shifting the burden of care from the patient to a coordinated ‘one-stop’ multidisciplinary framework can optimize the care cascade and offer a scalable strategy for achieving China’s HCV elimination targets.

## Methods

### Study design and participants

A non-randomized controlled intervention study was carried out in a cohort of 20,000 individuals with a history of substance addiction in Pudong New Area, Shanghai, China. The three models were implemented as follows: (1) the Self-Referral (SR) model: this model retrospectively examined individuals who tested positive for HCV antibodies during routine screening by the CDC in 2023. They were advised to independently seek confirmatory HCV RNA testing and treatment at hospitals; (2) the Standard Intensive Referral (SIR) model: this model reviewed antibody-positive individuals during routine screening by the CDC in 2024. They were provided with complimentary HCV RNA testing, and those confirmed positive were then encouraged to self-refer for treatment; (3) the Active Intensive Referral (AIR) model: an enhanced, proactive program collaboratively managed by the Pudong CDC, Zhongzhi Community Service Center, and partner hospitals, which provided centralized tracking, patient navigation, and facilitated linkage to care for antibody-positive individuals was carried out in 2024.

The study was approved by the Institutional Review Board of Shanghai East Hospital, School of Medicine, Tongji University (No.2023YS-022). The privacy and confidentiality of participants in the study are protected, including through data de-identification and restrictions on access to research data.

## Procedures

**Fig. 1:**
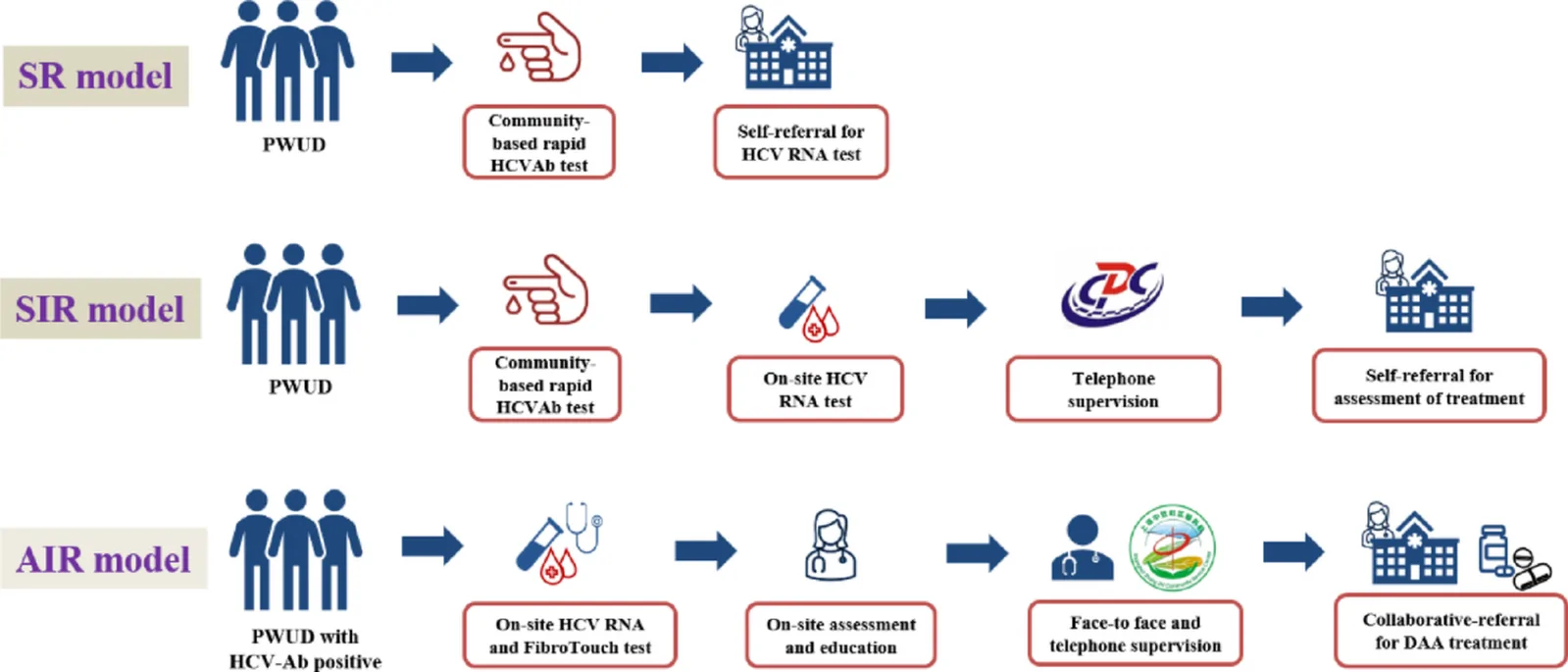
Simplified flowchart of the three modes. The self-referral (SR) model, the standard intensive referral (SIR) model, and the active intensive referral (AIR) model. HCV Ab, HCV antibody

Referral protocols were structured as follows across the three models. The Simplified flowchart was shown in Fig. 1.

In the SR model, community-based rapid HCV antibody testing was performed across 36 communities of Pudong New Area. Antibody-positive individuals received advice to self-present to a hospital for further nucleic acid testing and treatment.

Under the SIR model, antibody screening continued identically across 36 communities of Pudong New Area. However, antibody-positive individuals received complimentary on-site HCV RNA testing. Those with confirmed viremia were strongly encouraged to seek treatment at a designated hospital and received follow-up telephone calls from public health staff to promote treatment initiation.

The AIR model operates as a “closed-loop” ecosystem involving four key pillars. Pudong CDC provided overall program management, data monitoring, and coordination between community centers and hospitals. Zhongzhi Community Service Center was responsible for initial antibody screening and identifying high-risk individuals. Social Workers acted as the primary link between patients and clinicians. They administer baseline questionnaires (covering socio-demographics and HCV knowledge), facilitate centralized transportation, notify patients of results, and conduct biweekly follow-up calls. Specialist physicians in partner hospitals perform advanced diagnostics (HCV RNA, FibroTouch), prescribe DAA regimens, and manage clinical follow-up. Treatment adherence was monitored within the community via pill count.

It is worth noting that in the SR and SIR models, gender information was not retained during the retrospective data extraction process. HBsAg and Liver Stiffness (LSM) were not collected as part of the routine screening protocols in the SR and SIR models. These assessments were specific enhancements introduced in the AIR model. Genotypes were not obtained for the SR group, and 5 were missing in the SIR group because these models relied on hospital-based testing that was not systematically linked back to the study database.

## Study outcomes

The primary outcomes were HCV RNA detection rates and treatment initiation rates. Secondary outcomes encompassed the proportion of HCV RNA-reactive patients undergoing genotype assessment.

### Data analysis

Descriptive statistics characterized participant demographics and clinical profiles. The primary endpoints were HCV RNA detection rates among seropositive individuals and treatment initiation rates among viremic patients. A secondary endpoint included the proportion of HCV RNA-positive patients undergoing genotype testing. Between-group comparisons across the three referral models (SR, SIR, AIR) for categorical variables were performed using Pearson’s chi-square or Fisher’s exact tests, as appropriate. Continuous variables were compared using one-way ANOVA or nonparametric Kruskal–Wallis tests based on distributional assumptions. A two-sided *p*-value < 0.05 defined statistical significance. Multiple comparisons were addressed using the Bonferroni correction. All analyses were conducted using SPSS 21.0.

## Results

### Baseline characteristics

A total of 3926 PWUD in the SR model and 1973 PWUD in the SIR model underwent rapid HCV antibody testing. In the AIR model, 191 antibody-positive PWUD were enrolled for intensive evaluation. The median age across the three groups was 50.0 years (SR), 50.0 years (SIR), and 48.0 years (AIR) (*P* = 0.033), as shown in Table 1.

**Table 1:** Characteristics of patients HCV Ab+ve

Variable	SR model (*n* = 774)	SIR model (*n* = 916)	AIR model (*n* = 191)	*P* value
Median age (yrs) range	50.0(45.0,56.0)	50.0(45.0, 57.0)	48.0(43.0, 56.0)	0.033
Male gender n,%	Not Retained	Not Retained	161/191(84.3)	N/A
HCV RNA positive	70/146(47.9)	339/916(37.0)	74/191(38.7)	0.652
HBsAg positive	Not collected	Not collected	8/191(4.2)	N/A
LSM > 12.5 kPa n, (%)	Not collected	Not collected	52/191(27.2)	N/A

1. SR, the self-referral model; SIR, the standard intensive referral model; AIR, the active intensive referral model

2. The gender, data of HBsAg, and LSM were not obtained in the SR and SIR model

3. In the SR model, the denominator for RNA testing (*n* = 146) represents the 18.9% of antibody-positive individuals who successfully self-referred to a hospital; the remaining 628 individuals were lost to follow-up due to the lack of assisted referral protocols

Due to the retrospective nature of the SR and SIR cohorts, gender information was not retained for these groups. In the prospective AIR model, 84.3% of participants were male. Furthermore, clinical markers such as HBsAg status and liver stiffness measurement (LSM) were not collected under the routine protocols of the SR and SIR models. In the AIR model, 4.2% were HBsAg-positive, and 27.2% exhibited cirrhosis (LSM > 12.5 kPa) (Table 1).

## HCV testing and treatment initiation (three-group comparison)

**Fig. 2:**
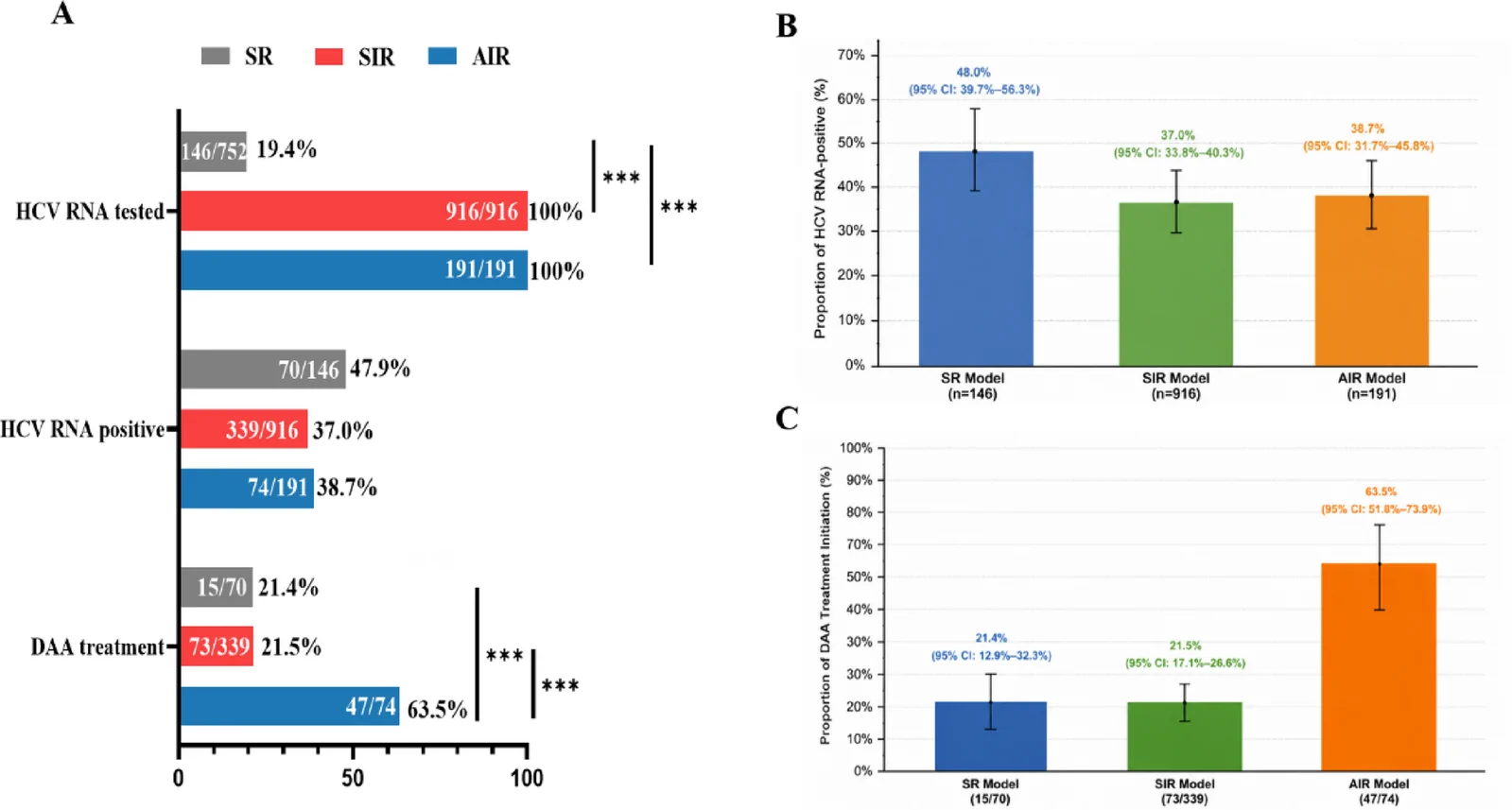
HCV testing, treatment initiation, and outcomes among the three models. **A** The primary care cascade outcomes. **B** The proportion of HCV RNA-positive and 95% CI. **C** The proportion of DAA treatment initiation and 95% CI. SR, self-referral; SIR, the standard intensive referral; AIR, the active intensive referral; DAA, direct-acting antivirals; CI: confidenceinterval. ***, *P* < 0.001

Comparisons across all three models were possible for primary care cascade outcomes (shown in Fig. 2). HCV RNA testing completion rates were 18.9% in the SR model, significantly lower than the 100.0% completion achieved in both the SIR and AIR models (*P* < 0.001) (Fig. 2A).

The proportion of individuals identified as HCV RNA-positive was 47.9% (70/146) (95%CI 39.9–56.1%) in the SR model, 37.0% (339/916) (95%CI 33.9–40.1%) in the SIR model, and 38.7% (74/191) (95%CI: 31.8–45.7%) in the AIR model (Fig. 2A and B). No significant difference in viremia prevalence was observed between the three groups (*P* = 0.652). However, DAA treatment initiation was significantly higher in the AIR model (63.5%, 95%CI 51.8–73.9%) compared to both the SIR (21.5%, 95%CI 17.1–26.6%) and SR (21.4%, 95%CI 12.9–32.3%) models (Fig. 2A and C, *P* < 0.001), representing a nearly threefold increase in linkage to care.

Further, we compared our results with the WHO stepwise model for the elimination of hepatitis C (as presented in Table 2), which better highlights the impact of this intervention throughout the entire clinical pathway.

**Table 2:** Alignment of Study Outcomes with the WHO HCV Elimination Cascade

WHO cascade Step	SR model	SIR model	AIR model	Gaps
Screen (Ab+)	774 identified	916 identified	191 identified	0.033
Confirm (RNA test)	18.9%	100%	100%	Primary leakage point in SR
Refer (RNA+)	70 viremic patients	339 viremic patients	74 viremic patients	N/A
Treat (DAA start)	21.4%	21.5%	63.5%	Secondary leakage point in SIR
Cure (SVR12)	Data not available	Data not available	Data not available	Future research target
Follow-up	Passive	Telephone only	Biweekly & Pill Counts	Optimization feature in AIR

SR, the self-referral model; SIR, the standard intensive referral model; AIR, the active intensive referral model

## Clinical and genotype profiles (SIR vs. AIR comparison)

Because genotype data were not obtained for the SR group, clinical and virological comparisons were restricted to the SIR and AIR models to ensure baseline comparability (Table 3).

**Table 3:** Characteristics of patient candidate to treatment between SIR and AIR models

Variable	SIR model (*n* = 339)	AIR model (*n* = 74)	*P* value
Median age (yrs) range	52.0(46.0, 60.0)	47.0(43.0, 57.0)	0.020
Male gender n, %	Not Retained	63/74(85.1)	N/A
Genotype^a^			0.354
GT1	75/334 (22.5)	13/74(17.6)	
GT2	3/334(0.9)	1/74(1.4)	
GT3	166/334(49.7)	38/74(51.4)	
GT3a	54/334(16.2)	15/74(20.3)	
GT3b	112/334(33.5)	23/74(31.1)	
GT6	79/334(23.7)	16/74(21.7)	

GT, genotype; HCV, Hepatitis C virus; LS, liver stiffness. SIR, the Standard Intensive Referral model; AIR, the Active Intensive Referral mode

a. There were 5 patients in the SIR model who had not completed the genotype testing

Among patient candidates for treatment, the median age was significantly lower in the AIR group (47.0 years) than in the SIR group (52.0 years) (*P* = 0.020). There was no significant difference in genotype distribution between the two models (*P* = 0.354). Genotype 3 remained the most prevalent in both cohorts, accounting for 49.7% of cases in the SIR model and 51.4% in the AIR model. Specifically, the 3b subtype predominated in both the SIR (67.5% of GT3) and AIR (60.5% of GT3) models.

### Participants who completed the questionnaire

In the AIR model, 181 participants completed the questionnaire. The results were shown in Table 4. Nearly 90% (89.1%) of the population had secondary education, with only one holding a university degree. In the case of marriage, less than half (47.5%) had stable marital relationships; Sixty-one of them were divorced, and 30 of them were unmarried. Hypertension affected 56 (30.9%) participants, and diabetes affected 19(10.5%) participants. More than 80% of the population is covered by health insurance.

**Table 4:** Participants who completed questionnaire

Variable	No.(%)
*Degree of education*	
Illiteracy	2/181(1.7)
Primary grades	10/181(8.3)
Secondary grades	107/181(89.1)
University degree	1/181(0.8)
*Marital status*	
Married	86/181(47.5)
Unmarried	30/181(16.6)
Divorced	61/181(33.7)
Widowed	2/181(1.1)
*Comorbid diseases*	
Diabetes	19/181(10.5)
Hypertension	56/181(30.9)
other	15/181(8.3)
*Insurance*	
Yes	151/181(83.4)
No	30/181(16.6)

**Fig. 3:**
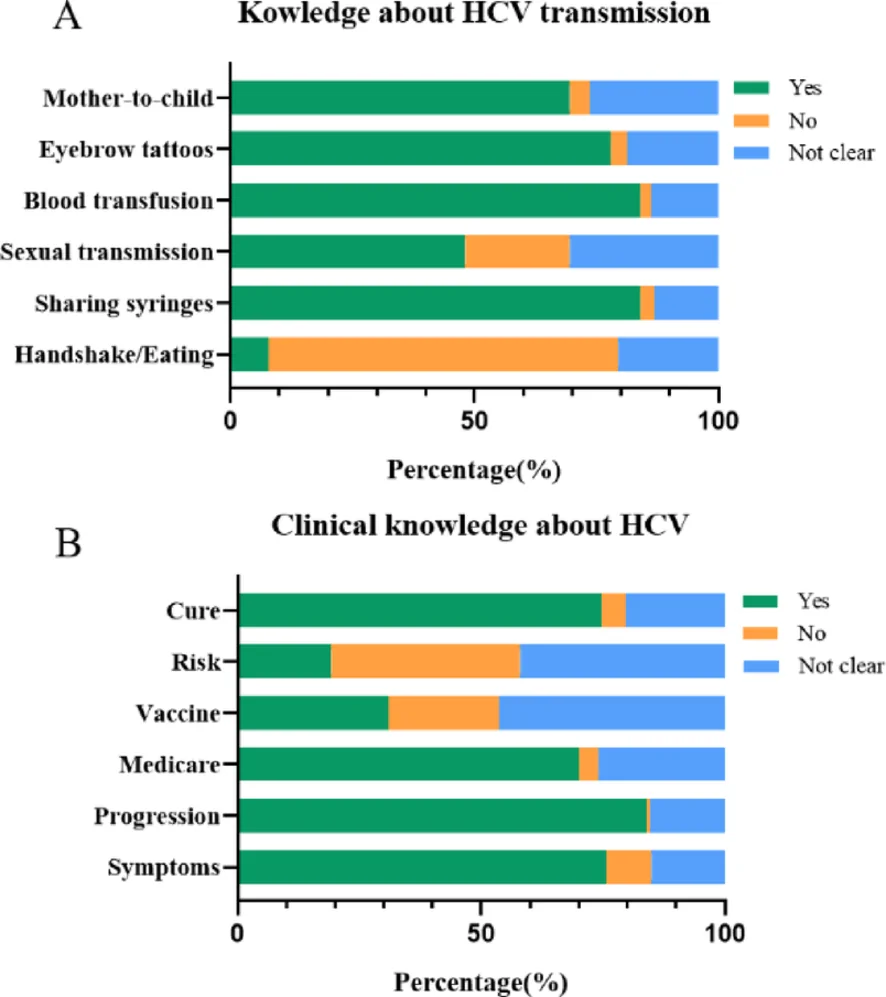
Knowledge of HCV among people who use drugs (PWUD) in the AIR model. **A** Knowledge of HCV transmission. Handshake/Eating: Can HCV be transmitted through handshaking or eating? Sharing syringes: Can HCV be spread by sharing syringes? Sexual transmission: Can HCV be transmitted sexually? Blood transfusion: Can you get the HCV by entering the blood of a person with HCV? Eyebrow tattoos: Is it possible to be infected with the HCV by getting eyebrow tattoos? Mother-to-child: Can a baby born to a pregnant woman with HCV be infected with HCV? **B** Clinical knowledge of HCV. Symptoms: Any symptoms with HCV patients? Progression: Can chronic HCV progress to cirrhosis and liver cancer? Are HCV antivirals covered by Basic Medical Insurance in China? Vaccine: Is there a vaccine for HCV? Risk: Do you think you are at risk of contracting HCV? Cure: Did you know that HCV can be cured?

HCV knowledge was also assessed, with results shown in Fig. 3A and B. In terms of transmission, more than 70% of people were aware that HCV cannot be contracted through shaking hands or eating together. Approximately 80% identified risks from shared syringes, blood transfusions, or tattoos. However, more than 50% of people believed that HCV could not be, or was uncertain, transmitted through sex. Clinically, over 70% knew HCV often lacks symptoms and risks progression to cirrhosis or liver cancer. Most people understood that antiviral drugs for HCV are insurance-covered and that HCV is curable. 42% perceived themselves at high infection risk.

## Discussion

The findings of this study highlight the critical differences among the SR, SIR, and AIR models for improving the HCV care cascade among PWUD in Pudong New Area, Shanghai. While three models demonstrated similar proportions of HCV RNA positivity (~ 37–48%), the AIR model substantially outperformed the SIR model and SR model in treatment initiation, with 63.5% of eligible patients starting DAA therapy compared to only 21.5% under the SIR model and 21.4% in the SR model. This threefold increase underscores the effectiveness of a multidisciplinary, community-based approach to linking patients to care.

### Addressing the care cascade: from passive to active referral

A significant limitation of the SR model was the high loss-to-follow-up rate during the transition from antibody screening to RNA confirmation. Out of 774 seropositive individuals, 81.1% (*n* = 628) failed to complete RNA testing. This attrition is directly attributable to the model’s reliance on independent patient action, which forced individuals to navigate complex healthcare systems while facing social stigma and logistical barriers [20, 21]. Because demographic and clinical data for these 628 individuals were not retained, we cannot exclude the possibility of attrition bias. Those who completed testing (*n* = 146) may represent a subset with higher health literacy or more severe symptoms, potentially explaining the slightly higher, though non-significant, RNA positivity rate in the SR group (47.9%) compared to the SIR group (37.0%). This massive drop-off underscores the necessity of the “one-stop” diagnostic services provided in the SIR and AIR models to ensure the care cascade does not collapse at the first referral step [22, 23].

However, the SIR model’s success ended at diagnosis. Despite being notified of their status, only 21.5% of SIR patients initiated treatment—a rate virtually identical to the SR model. This suggests that simply providing information and diagnostic access is insufficient [24, 25]. The AIR model’s success (63.5% initiation) was driven by its active navigation components, including dedicated transportation to hospitals, social worker coordination, and biweekly follow-up calls. These interventions appear to directly mitigate the social determinants of health identified in our questionnaire, such as low educational attainment and the lack of stable marital relationships, which are traditionally linked to poor health-seeking behavior [26, 27].

### Clinical and preventive implications: genotype 3b and cirrhosis

Another notable finding is the high prevalence of HCV genotype 3 [28], particularly subtype 3b, which accounted for more than half of cases in both models. Genotype 3 has been associated with poorer treatment responses and higher risks of liver disease progression compared to other genotypes [29, 30]. This emphasizes the need for timely diagnosis and treatment initiation in PWUD populations, especially given that nearly one-third of patients in the AIR model had advanced liver stiffness (> 12.5 kPa) at baseline. This high burden of silent cirrhosis underscores the value of the AIR model as a life-saving intervention. By tripling treatment initiation, the model reduces the long-term community burden of liver failure and hepatocellular carcinoma [31, 32].

### Methodological limitations and bias

Despite these promising results, the observed advantages of the AIR model must be interpreted with caution. The study’s non-randomized, partially retrospective design introduces inherent selection and attrition bias. Participation in the AIR model was voluntary, potentially introducing a “healthy volunteer” effect, where individuals with higher intrinsic motivation and health literacy were more likely to enroll.

Furthermore, structural stigma may have influenced data integrity [20, 33]. In the retrospective SR and SIR cohorts, critical variables such as gender, education, and insurance status were not retained. This lack of overlapping baseline variables precluded the use of Propensity Score Matching (PSM) to fully adjust for confounders. Specifically, the AIR group was significantly younger (median 47.0 years) than the SIR and SR groups (*P* = 0.020), which may have influenced their ability to navigate treatment. Additionally, the high loss-to-follow-up in the SR model (81.1%) means those who did complete testing might represent a subset with severe symptoms or higher health awareness, potentially skewing the RNA positivity rates.

### Policy implications and future research

From a policy perspective, our findings suggest that scaling up multidisciplinary models like AIR could support future policy formulations and may contribute to China’s efforts to reach its HCV elimination targets. Comparable successes in Australia and Germany have shown that decentralized, community-driven programs are the most effective way to reach PWUD [34, 35]. However, future implementation must include rigorous health economic evaluations to assess the cost-effectiveness of social worker-led navigation.

Moreover, this study was limited by the lack of follow-up data on sustained virologic response (SVR) and the treatment rate still falls short of the WHO’s treatment target. While the AIR model successfully linked patients to treatment, the final step of the WHO elimination cascade—cure—remains to be validated in this cohort. Future research should utilize prospective randomized trials to confirm these results and evaluate the long-term clinical and economic impact of intensive referral strategies.

Finally, we acknowledge that the replicability of this study may be limited. Although the core components of the AIR intervention are clearly defined, the overall study design, which integrates both retrospective and prospective cohorts, along with inherent variations in data collection procedures across different models, may make it challenging for other research groups to fully replicate this comparative analysis under identical conditions. Future studies with more standardized prospective designs are needed to confirm our findings.

## Conclusion

In summary, the findings of this study indicate that the Active Intensive Referral (AIR) model is associated with higher rates of initiation of hepatitis C treatment among people who use drugs (PWUD). Through multidisciplinary collaboration, social worker-led navigation services, and targeted solutions to logistical barriers, this model offers a potentially effective pathway to bridge the‘last mile’between community screening and hospital-based treatment. Despite limitations, the AIR model may represent a promising approach to improving the HCV care cascade among PWUD and could potentially contribute to China’s efforts to optimize cascade management for hepatitis C elimination among specific high-risk populations.

## Data Availability

The dataset supporting the conclusions of this article is available from the corresponding author on reasonable request.
